# Forgetting, Reminding, and Remembering: The Retrieval of Lost Spatial Memory

**DOI:** 10.1371/journal.pbio.0020225

**Published:** 2004-08-17

**Authors:** Livia de Hoz, Stephen J Martin, Richard G. M Morris

**Affiliations:** **1**Laboratory for Cognitive Neuroscience, Centre and Division of NeuroscienceUniversity of Edinburgh, Edinburgh, ScotlandUnited Kingdom

## Abstract

Retrograde amnesia can occur after brain damage because this disrupts sites of storage, interrupts memory consolidation, or interferes with memory retrieval. While the retrieval failure account has been considered in several animal studies, recent work has focused mainly on memory consolidation, and the neural mechanisms responsible for reactivating memory from stored traces remain poorly understood. We now describe a new retrieval phenomenon in which rats' memory for a spatial location in a watermaze was first weakened by partial lesions of the hippocampus to a level at which it could not be detected. The animals were then reminded by the provision of incomplete and potentially misleading information—an escape platform in a novel location. Paradoxically, both incorrect and correct place information reactivated dormant memory traces equally, such that the previously trained spatial memory was now expressed. It was also established that the reminding procedure could not itself generate new learning in either the original environment, or in a new training situation. The key finding is the development of a protocol that definitively distinguishes reminding from new place learning and thereby reveals that a failure of memory during watermaze testing can arise, at least in part, from a disruption of memory retrieval.

## Abstract 2

Teasing apart memory defects in animal models is not an easy task. A new protocol reveals that failure of memory can arise, at least in part, from a disruption of memory retrieval

## Introduction

For more than a century, the phenomenon of retrograde amnesia (RA)—the loss of memory for events that occur prior to a variety of precipitating brain insults—has provided the foundation for theories of memory consolidation and the locus of trace storage (McGaugh 1966; Davis and Squire 1984; Dudai and Morris 2000). However, RA may also reflect the inability of a memory system to access a trace—a failure of memory retrieval (Warrington and Weiskrantz 1968). This very dilemma was noted by Ribot (1883, p. 475) in his seminal discussion of RA:

*“Two suppositions are equally warranted, viz., that either the registration of the prior states has been effaced; or that the retention of the anterior states persisting, their aptitude for being revived by associations with the present is destroyed. We are not in a position to decide between these two hypotheses.”*

Studies of RA have favoured a memory-consolidation interpretation in instances in which systematic variation of the time interval between experience or training and the subsequent brain insult has revealed a temporal gradation of RA (Squire 1992). Computational models also point to the need for a rapid encoding and storage system, together with a slower interleaving mechanism that is thought to underlie systems-level consolidation and long-term storage in the cortex (e.g., McClelland et al. 1995). However, the existence of some amnesic patients with long, flat gradients of RA extending for years or decades into periods of their life when memory function was normal provided some of the first evidence that RA might be due to retrieval failure (Sanders and Warrington 1971). This perspective on RA was initially supported by studies indicating that, in the anterograde domain, impaired memory could be alleviated by partial cues (Warrington and Weiskrantz 1968). However, these observations were later construed as reflecting the operation of a separate memory phenomenon called priming (Graf et al. 1984). Several animal studies have also indicated that a variety of ‘reminder' treatments delivered prior to retention testing can realize the expression of lost memories (Gold et al. 1973; Miller and Springer 1973; Spear 1973; Gold and King 1974; Riccio and Richardson 1984; Sara 1999), but it is not easy to distinguish priming-induced memory from explicit recall and recognition in animal studies. Experimental resolution of the consolidation-versus-retrieval controversy has been notoriously difficult, and no consensus has been achieved. A key methodological issue, and the focus of the new technique described here, concerns the need to demonstrate that the memory observed after a reminder treatment results from the reactivation of an existing memory (Miller and Springer 1972), rather than a facilitation of new learning (Gold et al. 1973).

In studies of spatial memory using the watermaze, amnesia for the location of the escape platform in posttraining probe trials (PTs) has generally been interpreted as a failure of learning, consolidation, or storage (D'Hooge and De Deyn 2001). To investigate the alternative possibility of retrieval failure, we deliberately created conditions that should maximize the possibility of seeing such an effect. This involved training rats to find an escape platform in a specific location followed by partial lesioning of the hippocampus. We reasoned that this would weaken but not completely disrupt the memory of the correct location by damaging a subset of the ensemble of stored traces. The animals' memory was tested and observed to be undetectable. This same memory test provided, however, the opportunity to remind animals that escape from the water was possible via an escape platform in the correct or incorrect location. One hour later, the animals' memory was tested again. We observed that memory was now detectably above chance and was equally strong when the animals had previously been given correct or potentially misleading information about the current location of the platform. Additional control procedures, and the performance of other groups with sham or complete hippocampal lesions, established that the earlier failure of memory must have been due, at least in part, to retrieval failure.

## Results

**Figure 1:**
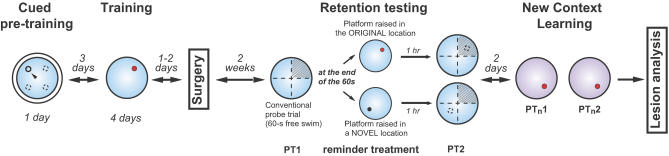
Experimental Design Outline of the different phases of testing. The platform position used during training is indicated by a red circle in the NE quadrant of the pool (large blue circle), although in practice platform locations were counterbalanced between NE and SW locations. The novel location, to which a subset of rats was exposed during reminding, is indicated by a black circle in the SW quadrant. This position was always opposite to that used during training. PT1 and PT2: probe test 1 and 2. The hatched areas represent the original training quadrant irrespective of the position of the platform (i.e., original or novel) during retention testing. PT_n_1 and PT_n_2: PTs during new context learning in the second pool.

A summary of the experimental design is provided in Figure 1 (see Materials and Methods).

### Training Prior to the Lesions

**Figure 2:**
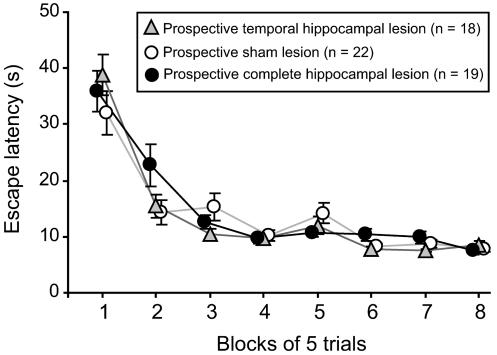
Training Mean latencies to escape from the water and climb onto the hidden platform during task acquisition. Data are averaged in blocks of five trials and grouped according to the lesion made at the end of training; note that all animals were unoperated during acquisition. Only rats that reached criterion (mean escape latency less than 15 s over the last ten trials) and whose lesions were considered acceptable (see Results: Surgery) are presented. Animals rapidly learned to locate the escape platform, and prospective lesion groups did not differ.

During cued pretraining, the rats quickly learned to search for, and climb onto, the visually cued escape platform. In the main spatial training phase, the animals rapidly learned to locate and raise the platform in order to escape from the pool (Figure 2), as indicated by the highly significant reduction in latencies over trials (*F*[7.78, 412] = 30.4, *p* < 0.001). Only animals that reached the acquisition criterion received lesions (69 out of 73 rats trained). The prospective lesion groups, trained as normal animals, did not differ (*F* < 1, *n* = 59; see Surgery below).

### Surgery

**Figure 3:**
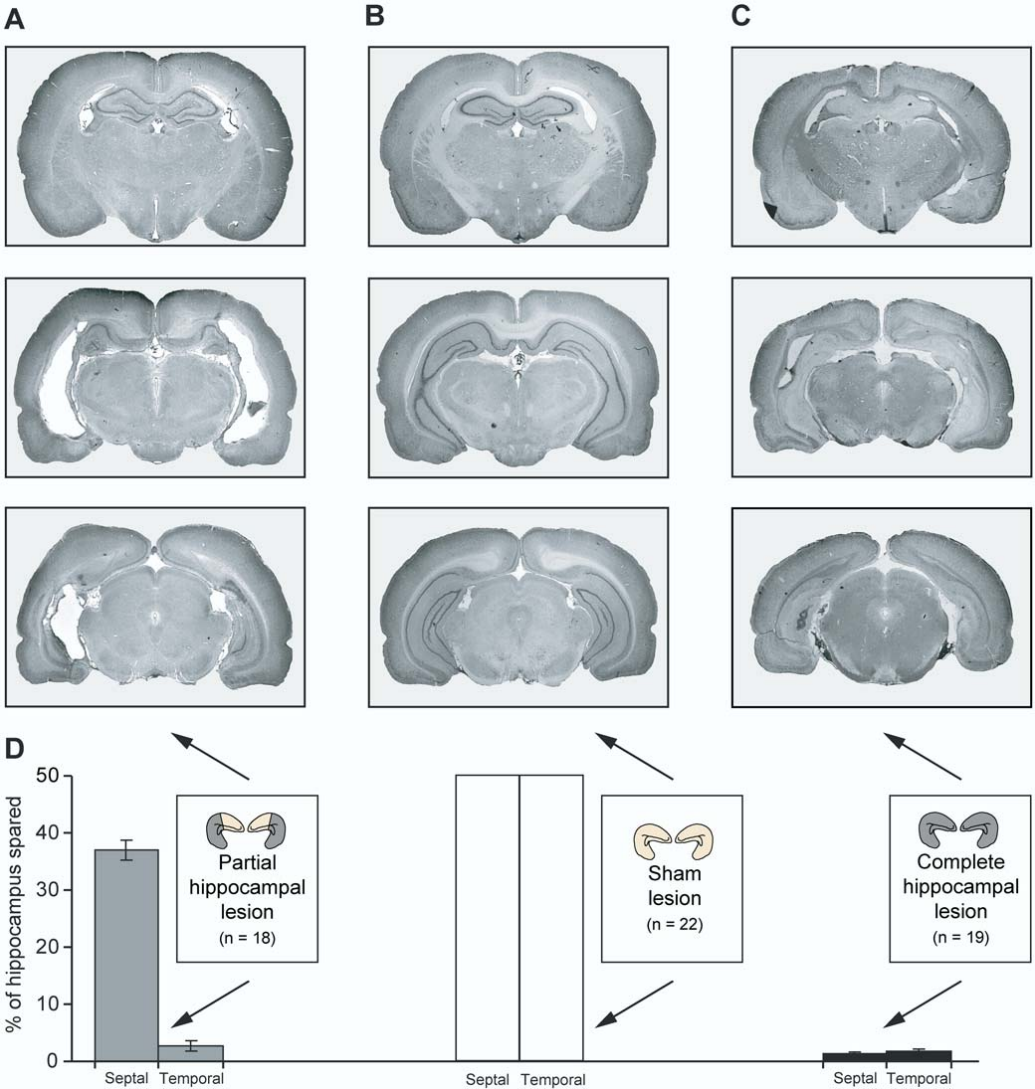
Lesion Analysis Representative photomicrographs of cresyl-violet-stained coronal brain sections taken from subjects belonging to each of the three lesion groups—partial hippocampal lesion (A), sham lesion (B), and complete hippocampal lesion (C). In each case, sections corresponding to anterior, middle, and posterior levels of the hippocampus are displayed. The mean area of spared hippocampal tissue in each group (see Materials and Methods for calculation) is plotted below in (D). Note that the volumes of spared tissue in the septal and temporal halves of the hippocampus are plotted separately, but these values are still expressed as percentages of the entire hippocampal volume—hence the value of 50% per half in shams. The cartoon hippocampi accompanying the graph indicate lesioned tissue in dark grey, and spared tissue in light cream. As intended, partially lesioned rats exhibited substantial sparing only in the septal (dorsal) half of the hippocampus, and rats with complete hippocampal lesions exhibited minimal sparing (less than 5% at either pole).

Of the 69 animals that received lesions, one died after surgery and nine were excluded based on strict histological criteria, leaving a total of 59 animals (22 sham lesions, 19 complete hippocampal lesions, and 18 partial hippocampal lesions; see Figure 3).

### Retention Testing

**Figure 4:**
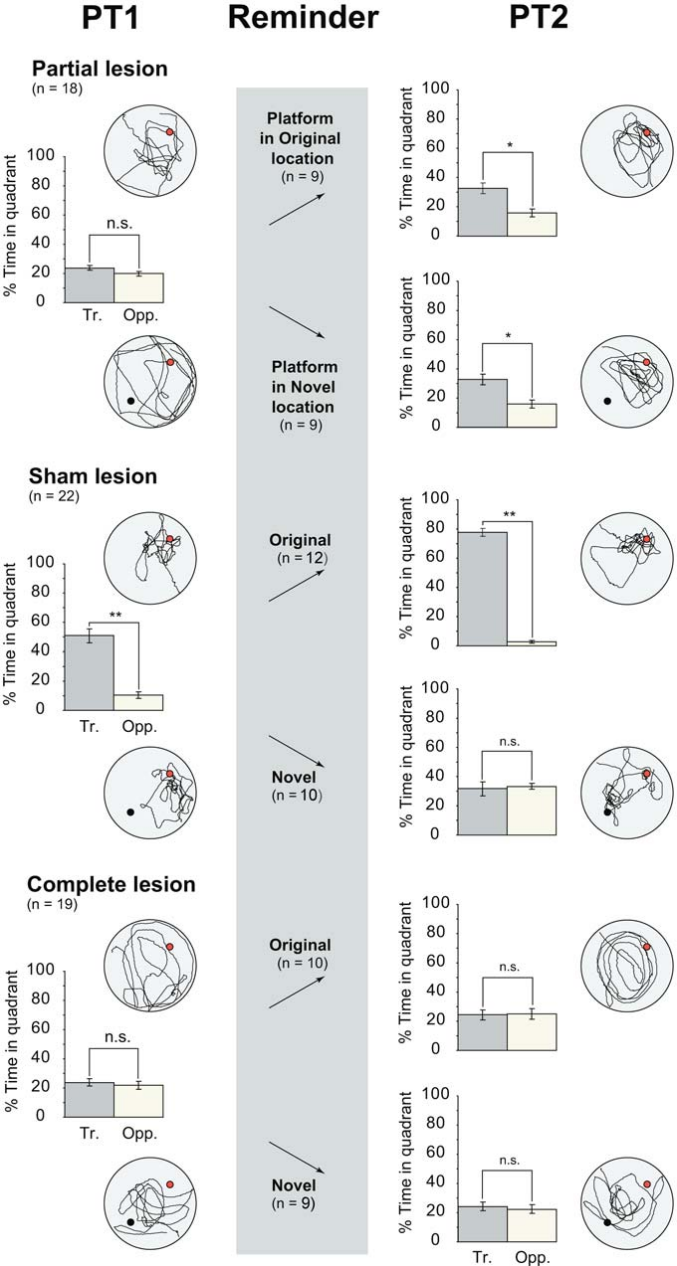
Retention Testing: Quadrant Analysis Percentage time during PT1 and PT2 spent in the training and opposite quadrants of the pool (left and right lanes) and the reminder treatment (grey central lane). The training location is represented as a red circle in the NE quadrant, and the novel location (novel subgroups only) as a black circle in the SW quadrant. In practice, NE and SW quadrants were counterbalanced. Rats with partial hippocampal lesions were unable to remember the platform location on PT1 but could be reminded of the training location by exposure, at the end of PT1, to a platform in the original or a novel location. (Note that the ‘reminder' lane simply refers to this exposure to a platform—PT1 is itself the ‘reminder trial.') The key finding is that the improvement in PT2 occurred irrespective of the platform location during reminding. In contrast, sham-lesioned animals exhibited some reversal learning upon exposure to the platform in a novel location. Complete-lesioned rats did not remember the platform location during either PT1 or PT2. *p < 0.05; **p < 0.01; n.s. = nonsignificant; comparisons with chance = 50%; one-sample t-tests. Representative swim paths are included.

**Figure 5:**
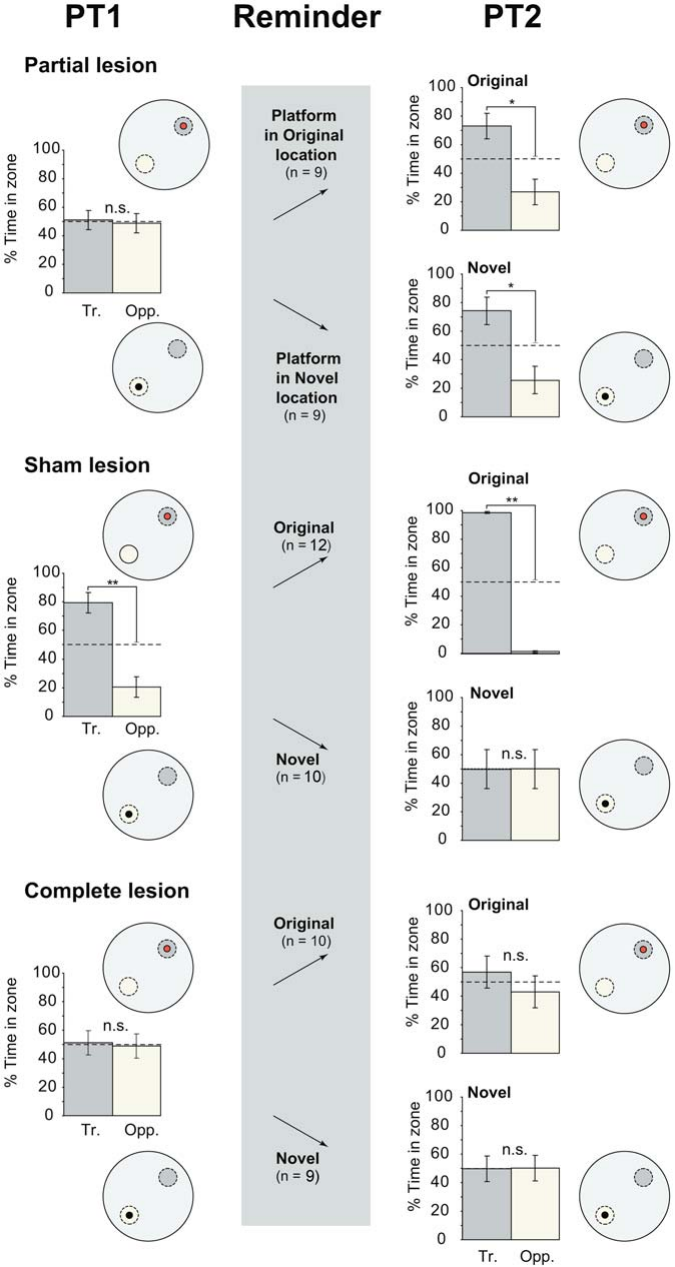
Retention Testing: Zone Analysis Percentage time in PT1 (left) and PT2 (right) spent within a zone, 20 cm in radius, centred on either the original training location (broken circle; grey) or an equivalent location in the opposite quadrant (broken circle; yellow), expressed as a percentage of the total time spent in both of the zones. The reminder treatment is again shown as the grey central lane and as the location where the hidden platform became available at the end of PT1 within these zones (original = red; novel = black). Consistent with Figure 4, rats with partial hippocampal lesions were amnesic in PT1 but could be reminded of the correct location, even by exposure to the platform in a novel location. *p < 0.05; **p < 0.01; n.s. = nonsignificant; comparisons with chance = 50%; one-sample t-tests.

The key new findings are shown in Figures 4 and 5 using two separate but related measures of memory retrieval: percentage time in quadrant (Figure 4) and a more sensitive measure, percentage time in a zone centred on the platform location (Figure 5; see Materials and Methods). An overall analysis of variance (ANOVA) of percentage time in the training (where the platform was located during training) and the opposite quadrants of the pool revealed a significant quadruple interaction (*F*[2, 53] = 7.66, *p* < 0.01) involving two between-subject factors: lesion group and platform location during the reminder treatment (original versus novel), and two within-subject factors: PT (PT1 and PT2) and quadrant (training versus opposite). In both figures, the initial memory expressed during PT1 is shown in the left lane. This reveals that the partially lesioned rats were at chance, whereas the sham-lesioned rats could remember the location of the platform (*t* = 6.15, *df* = 21, *p* < 0.005, paired-sample t-test, training versus opposite quadrant). The complete-lesioned animals were at chance. Analysis of percentage time in zone (Figure 5) likewise confirmed that memory was detectable in the sham lesion group (*t* = 4.18, *df* = 21, *p* < 0.005, one-sample t-test, comparison with chance = 50%), but not in the two lesion groups.

PT1 ended with the animals finding the platform in the original training location, or in a novel location in the ‘opposite' quadrant of the pool (middle lane in Figures 4 and 5; see Materials and Methods for explanation of terminology). These different events at the end of the swim trial potentially served both as a reminder of what happens in a watermaze, namely, escape from the water at a particular location, and/or as an opportunity for new learning. We reasoned that if the reward of escaping from the water served only to support new learning, animals capable of learning would show an enhanced bias towards the training location after finding the platform in the original location, but a reduced bias after finding it in the opposite novel location. Conversely, if these events served only as reminder cues, they might be equally effective in reminding the rats of the original training location.

The key new finding is that the partial lesion group displayed a bias for the training quadrant that was equivalent whether the animals had found the platform in the original training location or in the novel opposite location, at the end of PT1. The overall ANOVA of the PT2 quadrant data revealed a triple interaction of lesion group × quadrant (training versus opposite) × platform location during reminding (novel versus original) (*F*[2, 53] = 19.28, *p* < 0.001). With respect to the performance of the partial lesion group alone on this quadrant measure (see Figure 4, right lane), there was a significant improvement between PT1 and PT2 (*F*[1, 16] = 7.98, *p* < 0.02) and no difference between novel and original reminding locations (*F* < 1). The partial lesion group also showed a highly significant preference for the training quadrant versus the opposite quadrant on PT2 (*F*[1, 16] = 16.83, *p* < 0.001). The same pattern of results is apparent in the zone data (see Figure 5) where, overall, the partial lesion group displayed a significant improvement between PT1 and PT2 (*F*[1, 16] = 7.64, *p* < 0.02) that also did not differ between ‘novel' and ‘original' groups (*F* < 1). Because a bias for the training location appeared even in the animals that were exposed to a novel platform position, memory on PT2 cannot be attributed to relearning of the platform location.

In contrast, sham-lesioned animals behaved quite differently in PT2 as a function of whether the platform was presented in the original or the novel location during the reminder treatment. Performance showed a further bias towards the training location between PT1 and PT2 following the event of climbing onto the escape platform in its original location, but exposure to the novel location resulted in a reduction in time spent in the training zone—a partial reversal. Supported by significant interactions in the overall ANOVA, analysis of time spent in the training quadrant revealed that, as expected, sham-lesioned animals reexposed to the original location increased their time there between PT1 and PT2 (*F*[1, 11] = 12.41, *p* < 0.005). Conversely, sham-lesioned animals exposed to the novel location exhibited modest reversal learning, increasing their time in the opposite quadrant (*F*[1, 9] = 9.35, *p* < 0.02). The same pattern of results was obtained from the analysis of time in the training zone (Figure 5), for which a significant interaction between PT (PT1 or PT2) and platform location during reminding (original versus novel) was observed (*F*[1, 20] = 5.46, *p* < 0.05).

Complete-lesioned rats performed at chance during all PTs (see Figures 4 and 5, left and right lanes). That is, their behaviour during the retention tests before and after the reminder treatment showed no impact of that treatment.

### Novel Context Learning

**Figure 6:**
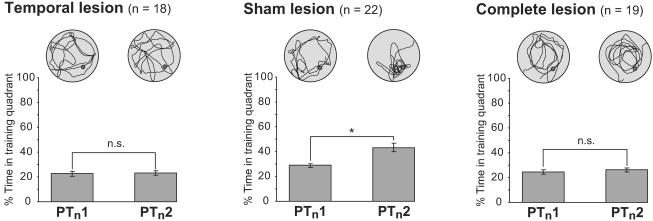
Novel Context Learning Percentage time spent in the target quadrant containing the escape platform during one-trial new learning in a different pool. *p < 0.05; n.s. = nonsignificant; comparison of percentage time spent in training zone during PT_n_1 and PT_n_2; paired-sample t-tests. New learning was observed only in sham-lesioned rats.

As an independent test of whether the reminder treatment of escape onto a platform could support new learning, all animals were taken to a second (‘downstairs') watermaze and given two PTs (Figure 6). This was a novel environment, and, therefore, there was no reason to expect the animals to perform at better than chance levels in the first of these PTs in a novel environment (PT_n_1). However, escape from the water at the end of this PT might be sufficient to support new one-trial learning. Such learning was absent in the partial hippocampal lesion group (*F* < 1). The sham lesion group, in contrast, did learn (*F*[1, 21] = 4.51, *p* < 0.05), performing significantly better than the lesioned groups on PT_n_2 (post hoc Ryan–Einot–Gabriel–Welsch range test, *p* < 0.005). The complete lesion group again showed no evidence of learning in a new environment (*F* < 1).

## Discussion

The key finding of this study is that rats with partial lesions of the hippocampus can be reminded of a preoperatively learned escape location in a watermaze by both correct and potentially misleading information. Whereas sham-lesioned rats showed new one-trial learning towards or away from the originally trained quadrant as a function of the type of reminder treatment to which they were exposed, partially lesioned animals were unable to learn. Instead, the first PT served only as a reminder of the original platform location irrespective of where in the pool the platform was raised at the end of this trial. Rats with complete hippocampal lesions showed neither new learning nor reminding.

There is an extensive classic literature on the nature and effectiveness of reminder treatments (Riccio and Richardson 1984). Exposure to the training context, noncontingent stimuli, or additional training trials are just some examples of methods successfully used to remind animals of a prior training experience (Zinkin and Miller 1967; Miller and Springer 1973; Mactutus et al. 1979; Gisquet Verrier and Schenk 1994; Przybyslawski and Sara 1997). Controversy did, however, surround studies that interpreted memory following a reminder treatment as evidence that the original amnesia was the result of a retrieval deficit (Zinkin and Miller 1967; Miller and Springer 1973). It was argued that a reminding trial simply strengthens a weak memory that is behaviourally unobservable, similar to what happens during initial learning (Cherkin 1972; Gold et al. 1973; Haycock et al. 1973; Gold and King 1974), or that, when amnesia is complete, it results in one-trial learning or response generalization. However, manipulations that are unlikely to produce new learning can also serve as effective reminders. Examples include pharmacological manipulations of the internal state (Mactutus et al. 1980; Concannon and Carr 1982) and reexposure to the amnestic agent prior to retention testing (Thompson and Neely 1970; Hinderliter et al. 1975). In many such studies, however, the use of inhibitory avoidance as a memory test makes it difficult to determine the cognitive ‘content' (cf. Riccio and Richardson 1984) of the behaviour expressed during retention testing. Although memory reactivation may have occurred when a rat inhibits movement that previously led to electric shock, an alternative interpretation is that a generalized fear state has been induced. The issue of whether and when amnesia reflects a storage or retrieval deficit was, thus, left unresolved.

Two features are distinctive about our study. First, unlike in many previous studies, the reactivated memory involves the recall and expression of highly specific information—a discriminable position in space, and not just a faster escape latency, or greater freezing. Second, despite exposure to a novel platform location leading to reversal learning in the sham lesion group, the partial lesion group displayed only reminding of the original platform location. This distinction is important because, with the current revival of interest in memory retrieval, our protocol circumvents the ambiguities involved in the use of relearning as an index of retention. One example of a study that used a reacquisition rather than a true reminding protocol (Land et al. 2000) revealed that a reminder prior to retention testing could alleviate amnesia in animals with hippocampal lesions. However, it is difficult to distinguish between ‘pure' reminding and the facilitation of new learning using reacquisition alone.

Nonetheless, the watermaze task is deceptively complex, and successful performance depends on the operation of several distinct memory systems (Bannerman et al. 1995; Whishaw and Jarrard 1996; Warburton and Aggleton 1999; Eichenbaum 2000; White and McDonald 2002). Accordingly, while no new learning of the platform location occurs in the partial and complete lesion groups, some ‘procedural' learning may take place during PT1; this may enhance a weak, subthreshold spatial memory to a point at which it can be expressed in PT2. However, for this argument to be plausible, one would expect there to be minimal retention of the procedural components in PT1. This was clearly not the case, as rats with both partial and complete hippocampal lesions did not behave like naïve animals during PT1. They searched at an appropriate distance from the pool walls and readily climbed onto the escape platform when it was eventually made available. Procedural learning is also generally well retained over time and, being slow, unlikely to change much in one trial. We also doubt that the recovery of memory on PT2 reflects the emergence of latent memory mediated solely by an extrahippocampal structure, but not expressed during PT1. For example, rats with complete hippocampal lesions have been shown to learn a spatial conditioned-cued preference mediated by the amygdala (White and McDonald 1993), a form of memory that is partially masked by hippocampus-dependent learning in normal rats (McDonald and White 1995). However, seeing reminding in partial but not complete hippocampus-lesioned animals argues against this possibility in this case. Finally, the recovery of a simple stimulus–response strategy based on approaching single cues is unlikely, as novel start locations were always used during retention testing (cf. Eichenbaum et al. 1990; see Materials and Methods). Under these circumstances, it is reasonable to interpret the apparently complete amnesia observed in PT1 as, at least in part, a failure of spatial memory retrieval.

Our use of partial hippocampal lesioning introduces several other issues. First, it is a technique that is arguably more relevant to human amnesia, in which damage to a structure is typically incomplete. Second, it is also relevant to the many studies in which a pharmacological intervention is applied at a single site within a brain region—microinfusion into the dorsal hippocampus, for instance, is likely to have minimal effects on ventral hippocampal tissue (see Steele and Morris 1999). Third, and perhaps most interesting, is the question of where memory traces are located. Given that reminding only occurs in partially lesioned rats, it is reasonable to suppose that spatial memory traces are either located (and reactivated) within the hippocampus, or that the hippocampus is required for the process of reactivation or expression of a reactivated memory stored elsewhere. According to the latter hypothesis, spatial memory traces might be stored in cortex but require fast synaptic transmission in the hippocampus to be retrieved (cf. Teyler and DiScenna 1986)—at least during the period after training and before the completion of systems-level consolidation. Alternatively, some hippocampal tissue might be required for cortically expressed memory to gain access to striatal motor planning and executive systems. Findings reported by Virley et al. (1999) suggest that this retrieval hypothesis might not be implausible. In this study, monkeys with CA1 pyramidal cell lesions were amnesic for a preoperatively acquired visuospatial discrimination. Subsequent grafting of CA1 pyramidal cells resulted in the recovery of memory for a second preoperatively acquired discrimination. As the grafted tissue cannot contain specific memory traces, the implication is that the recovery of some aspects of CA1 cellular function is sufficient for the information processing mediating the retrieval of memories stored elsewhere.

In raising many more questions than they answer, the present findings open a potential avenue of research into the neural dynamics of memory reactivation and retrieval. Specific interventions such as local AMPA receptor blockade (cf. Riedel et al. 1999) might be directed at the hippocampus or cortex during PT1 or PT2. Such a study could provide information about the role of these structures—and their network interactions—in the reactivation of apparently lost memories, and in their subsequent retrieval. For example, hippocampal neural activity may be necessary for effective retrieval, but perhaps not for the reminding-induced reactivation of memory, even for an ostensibly hippocampus-dependent task (cf. Land et al. 2000). Similarly, the necessity for hippocampal neural activity during retrieval might vary as a function of time after memory consolidation. In addition, the determinants of the reminder phenomenon itself remain unclear. It would be useful to establish whether reinforcement in the form of an escape platform is, in fact, necessary during PT1, or indeed whether a reminder trial in a separate pool would have been effective. Experiments involving partial versus complete sets of cues might also provide valuable insights into the reminding process (cf. O'Keefe and Conway 1978). These and related analyses will be the subject of future studies.

Dissociating the storage and retrieval functions of the hippocampus in memory is central to our understanding of the role of hippocampo–cortical connections. Many theories of hippocampal function are based on the idea that the hippocampus acts as a mediating link between different cortical regions during the interval before systems consolidation is complete (Teyler and DiScenna 1986; Squire and Alvarez 1995). Paradoxically, the same features that point to the alternative possibility—that the hippocampal formation is a site of encoding and long-term storage of complex multimodal memories within its distributed intrinsic circuitry (Moscovitch and Nadel 1998)—also place this group of structures in an ideal position to help reactivate memories from traces distributed over several cortical structures, perhaps via a mechanism such as pattern completion (see Marr 1971; Nakazawa et al. 2002). It is possible that, when the hippocampus is partially damaged and the cortico–hippocampal network is therefore degraded, retrieval is only possible once a more complete recreation of the training situation, possibly including reexposure to a platform, is provided. Although comparisons across different species and forms of memory should be viewed with caution, this scenario is reminiscent of Tulving's encoding specificity principle (Tulving and Pearlstone 1966; Thomson and Tulving 1970) in that exposure to similar cues during encoding and retrieval phases permits the recovery of the original memory, despite the provision of incorrect information about the target location itself. Paradoxically, the poor learning abilities of partially lesioned rats might explain why a trial ending with exposure to a novel spatial location can serve as a reminder for the original location—by limiting new learning of the new location, a reactivated memory for the old location is unmasked.

## Materials and Methods

### 

#### Subjects

We used a total of 73 male Lister Hooded rats obtained from a commercial supplier (Charles River Laboratories, United Kingdom). They were pair-housed in plastic cages with sawdust bedding and ad libitum access to food and water. Their care and maintenance and all experimental procedures were carried out in accordance with United Kingdom Home Office Regulations.

Behavioural testing was conducted using two separate circular pools, 2.0 m in diameter and 60 cm high, each located in well-lit rooms with numerous distal visual cues. One pool was used for training and retention (‘upstairs') and the other for new context learning (‘downstairs'). The pools were filled with water at 25 °C ± 1 °C made opaque by the addition of 200 ml of latex liquid (Cementone-Beaver, Buckingham, United Kingdom). We used the ‘Atlantis platform' (Spooner et al. 1994), a polystyrene platform that becomes available by rising from the bottom of the pool only if the animals swim to and stay within a specified ‘dwell radius' centred on the correct location for a predetermined ‘dwell time.' When risen, the top of the platform remained 1.5 cm below the water surface. The animals' swimming was monitored by an overhead video camera connected to a video recorder and an online data acquisition system (Watermaze, Watermaze Software, Edinburgh, United Kingdom; Spooner et al. 1994) located in an adjacent room. This system digitizes the path taken by an animal and computes various parameters such as escape latency, time spent in a zone overlying the platform, and other conventional measures of watermaze performance.

#### Training protocol

Testing was carried out according to the schedule illustrated in Figure 1.

#### Cued pretraining

This phase consisted of a single day of nonspatial cued training in the ‘upstairs' watermaze (curtains drawn around the pool to occlude extramaze cues, with ten trials in two sessions of five trials each (intertrial interval ≈ 20 min; intersession interval ≈ 3 h). The visible cue was suspended approximately 25 cm above the platform, which was moved every two trials to one of four possible locations, according to a pseudorandom schedule; the dwell radius was set at 20 cm, and the dwell time was 1 s.

#### Training

Training on a spatial reference memory task began 3 d later in the same watermaze. Rats received ten trials/day, in two sessions of five consecutive trials each (intersession interval ≈ 2 h), for 4 d. The dwell time was set to 0.5 s throughout training, but the dwell radius was gradually reduced over days (day 1: 20 cm; day 2: 15 cm; days 3 and 4: 13 cm). This schedule was intended to promote accurate and focused searching, but without generating the highly perseverative strategy that typically results from the use of long dwell times (Riedel et al. 1999). Rats were given a maximum of 120 s to find an escape platform located at the centre of either the NE or SW quadrant, after which they remained on the platform for 30 s On the rare trials in which a rat failed to escape within 2 min, the experimenter placed a hand above the correct location in order to guide the animal to the platform. For each animal, the platform position remained constant throughout training, but start locations (N, S, E, or W) were varied pseudorandomly across trials. Only those animals achieving the acquisition criterion of mean escape latencies of 15 s or less on day 4 of training proceeded to the next phase of testing.

#### Surgery

Surgery took place 1–2 d after the end of training. Rats were given either partial or complete bilateral neurotoxic lesions of the hippocampal formation (DG and CA fields), or sham surgery. Complete lesions were intended to remove 85% or more of the total hippocampal volume. Partial lesions targeted the temporal two-thirds of the hippocampus, sparing the septal (dorsal) third of the structure. The rats were assigned to groups of equivalent mean performance on the basis of their escape latencies during the final day of training. Lesions were made with ibotenic acid (Biosearch Technologies, Novato, California, United States; dissolved in 0.1 M phosphate-buffered saline [pH 7.4] at 10 mg/ml) following the protocol of Jarrard (1989). The animals were anaesthetized with an intraperitoneal injection of tribromoethanol (avertin) and placed in a Kopf Instruments (Tujunga, California, United States) stereotaxic frame such that Bregma and Lambda lay on the same horizontal plane. Rats received nine or 13 injections of ibotenic acid (partial and complete lesion groups, respectively; 0.05 μ1, 0.08 μ1, or 0.1 μ1 per injection) at different rostrocaudal and dorsoventral levels via an SGE syringe secured to the stereotaxic frame (see de Hoz et al. 2003). The injection rate was 0.1 μ1/min, and the needle was removed very slowly 90 s after the injection. A total of 0.65 μ1 or 0.91μ1 per hemisphere was necessary for the partial and complete lesions, respectively. The coordinates were modified from Jarrard (1989) to suit the slightly different brain size of Lister Hooded rats and to achieve the desired amount of partial hippocampal damage (see de Hoz et al. 2003). Sham lesions were made in the same way, with the injections replaced by a piercing of the dura (intended to cause comparable neocortical damage).

#### Retention testing

This phase began 14 d after the end of training. It consisted of two PTs (PT1and PT2) spaced 1 h apart, with a reminder treatment occurring at the end of PT1.

Each PT (PT1 and PT2) began with a standard 60-s swim with the platform unavailable. In each PT, the rats were placed into the pool in either the adjacent right or the adjacent left quadrants with respect to the training quadrant. Start positions were counterbalanced across PTs and across rats. At the end of the 60 s the platform was raised and the animals were allowed to find and climb onto it. The rats were allowed a further 60 s to locate the platform once risen (but still hidden just below the water surface); if unsuccessful within this period, they were guided to the platform. They then remained on the platform for 30 s.

The raising of the platform at the end of PT1 constituted the reminder treatment; thus PT1 is sometimes referred to as the ‘reminder trial.' A key variable was that the platform was raised in either the original training location (half the animals) or in a novel location in the centre of the opposite quadrant of the pool (the other half). Note that reminding using the original location always occurred in the training quadrant, and reminding using the novel location always occurred in the opposite quadrant. However, whereas the terms ‘training' and ‘opposite' are used to refer to physical areas of the pool, ‘novel' and ‘original' refer also to separate groups that received each type of reminder.

For analysis of the different behavioural phases, several measures of performance were assessed, including escape latency, swim speed, and time spent within defined regions of the pool. Memory retention during PTs is inferred from the time spent in each quadrant of the pool as a percentage of the 60-s duration of the PT. A more sensitive measure can be obtained by analysing percentage time spent within a specified radius (zone) centred on the platform location (Moser and Moser 1998). When time in zone is presented, it is expressed as a percentage of the total time spent in both the original training zone and the novel opposite zone. Statistical analysis (SPSS, Chicago, Illinois, United States) began with an ANOVA followed by appropriate post hoc comparisons. Numerical data are reported as mean ± standard error (s.e.m.) throughout.

#### Novel context learning

New learning was assessed the next day in a separate ‘downstairs' watermaze that constituted a novel context. The protocol was identical to that used during ‘upstairs' retention testing, i.e., two rewarded PTs (PT_n_1 and PT_n_2) spaced 1 h apart.

#### Lesion analysis

At the end of behavioural testing, rats were perfused intracardially with saline followed by 10% formalin under terminal pentobarbitone anaesthesia (Euthatal, 1 ml). Their brains were removed and stored in 10% formalin for 24 h before being blocked and embedded in egg yolk. The embedding procedure is described in de Hoz et al. (2003). Coronal, 30-μm sections through the hippocampus and other structures were cut using a cryostat: every fifth section was recovered, mounted on a slide, and stained with cresyl violet (see Figure 3A–3C).

The relative volume of spared tissue was calculated by measuring the area of hippocampus spared in each section of a particular brain according to the following protocol: Each coronal section containing hippocampus was placed under a photomacroscope (Wild, Heerbrugg, Switzerland), and the image taken by a mounted video camera was imported into NIH Image 1.63 (National Institutes of Health, Bethesda, Maryland, United States). The area of spared hippocampal tissue in each section was then outlined and automatically calculated. Surrounding fibres such as the fimbria were excluded on the grounds that they would not be considered in a section were all the hippocampal cells dead. The sections were spaced 150 μm apart, yielding up to 32 sections in a sham lesion animal, and fewer in animals with acceptable partial lesions. For each rat, the total hippocampal ‘volume' was calculated by adding the area of hippocampal tissue spared in each successive section. The proportion of hippocampus spared for each lesioned animal was expressed as a percentage of the mean hippocampal ‘volume' for sham-lesioned animals. Values for the left and right hippocampi were initially calculated separately and then averaged (see Figure 3D).

Strict criteria for acceptance of a lesion were used. The lesion had to be confined to the hippocampus in all cases, and leave intact tissue volumes of 25%–50% in the septal hippocampus with minimal sparing (less than 10%) elsewhere in the structure in the case of partial lesions, or less than 15% total hippocampal sparing in the case of complete lesions. Animals with minimal subicular damage, typically located at medial levels of the structure, were accepted.

## Abbreviations

ANOVA: analysis of variance
PT: probe trial
PT_n_: probe trial in a novel environment
RA: retrograde amnesia
